# Epithelial atrophy in oral submucous fibrosis is mediated by copper (II) and arecoline of areca nut

**DOI:** 10.1111/jcmm.12622

**Published:** 2015-08-06

**Authors:** Imran Khan, Ila Pant, Sivakrishna Narra, Rekha Radhesh, Kannan Ranganathan, Somanahalli Girish Rao, Paturu Kondaiah

**Affiliations:** a Department of Molecular Reproduction, Development and Genetics, Indian Institute of ScienceBangalore, India; b Clumax DiagnosticsBangalore, India; c Department of Oral Pathology, Ragas Dental College and HospitalChennai, India; d Department of Oral and Maxillofacial Surgery, D.A. Pandu Memorial - R.V. Dental College and HospitalBangalore, India

**Keywords:** areca nut cytotoxicity, copper, epithelial atrophy

## Abstract

Exposure of oral cavity to areca nut is associated with several pathological conditions including oral submucous fibrosis (OSF). Histopathologically OSF is characterized by epithelial atrophy, chronic inflammation, juxtaepithelial hyalinization, leading to fibrosis of submucosal tissue and affects 0.5% of the population in the Indian subcontinent. As the molecular mechanisms leading to atrophied epithelium and fibrosis are poorly understood, we studied areca nut actions on human keratinocyte and gingival fibroblast cells. Areca nut water extract (ANW) was cytotoxic to epithelial cells and had a pro-proliferative effect on fibroblasts. This opposite effect of ANW on epithelial and fibroblast cells was intriguing but reflects the OSF histopathology such as epithelial atrophy and proliferation of fibroblasts. We demonstrate that the pro-proliferative effects of ANW on fibroblasts are dependent on insulin-like growth factor signalling while the cytotoxic effects on keratinocytes are dependent on the generation of reactive oxygen species. Treatment of keratinocytes with arecoline which is a component of ANW along with copper resulted in enhanced cytotoxicity which becomes comparable to IC_50_ of ANW. Furthermore, studies using cyclic voltammetry, mass spectrometry and plasmid cleavage assay suggested that the presence of arecoline increases oxidation reduction potential of copper leading to enhanced cleavage of DNA which could generate an apoptotic response. Terminal deoxynucleotidyl transferase dUTP Nick End Labeling assay and Ki-67 index of OSF tissue sections suggested epithelial apoptosis, which could be responsible for the atrophy of OSF epithelium.

## Introduction

Oral submucous fibrosis (OSF) is a lesion that is characterized by chronic inflammation and epithelial atrophy along with loss of rete ridges leading to hyalinization and fibrosis of submucosal tissue. This condition has been considered as a precancerous lesion with a higher probability of developing oral squamous cell carcinoma. At later stages of the disease, patients experience difficulty in opening the mouth due to excessive fibrosis of the buccal mucosa. Areca nut chewing habit has been proposed as a major etiological factor for the OSF pathogenesis 1. Exposure of oral cavity to areca nut induces several physical, biochemical and molecular changes, leading to pathological conditions such as OSF or oral cancer. This was also supported by the experiments in mice where in, a long-term topical application of areca nut aqueous extract resulted in epithelial atrophy followed by infiltration of immune cells into the sub-epithelial layer and gradual deposition of collagenous matrix components in the sub-epithelial region 2, leading to OSF. However, molecular mechanisms underlying areca nut induced changes in oral epithelium and fibroblasts in the manifestation of OSF are poorly understood. Both the epithelium and the underlying fibroblast cells are the primary targets of areca nut constituents namely alkaloids and polyphenols. Areca nut extracts and arecoline have been shown to be cytotoxic to both epithelial and fibroblast cells but a corresponding cell death has never been shown in OSF tissues 3–5. Cytotoxicity induced by arecoline has been shown to be reactive oxygen species (ROS) mediated which is induced by the suppression of catalase activity in the epithelium 4. Arecoline has been proposed to be responsible for areca nut mediated actions on epithelial cells but at a relatively higher concentration compared to areca nut extract 6. Therefore, it is highly probable that other components in areca nut, in addition to arecoline are important in some of the areca nut mediated effects relevant for epithelial cytotoxicity. In addition to alkaloids, areca nut is known to possess higher levels of metals, the most abundant being copper 7,8. Higher levels of copper have been observed in OSF tissues with a concentration gradient varying from being higher in the epithelium to lower in the connective tissue 9. As presence of metal could add on to the areca nut cytotoxic actions on epithelial cells, we hypothesized that areca nut induced cytotoxicity of epithelial cells could be due to a combination of alkaloids and copper which may lead to epithelial atrophy, a hallmark of OSF.

Interestingly, salivary concentration of predominant alkaloid arecoline along with its N-nitrosoamines derivatives reaches to toxic levels in areca nut chewers 10. Therefore, to elucidate the possible mechanisms of epithelial atrophy in OSF, we studied the mechanism of cytotoxicity and proliferation induced by areca nut constituents in epithelial and fibroblast cells, respectively. In addition, by immunohistochemistry (IHC), we studied the status of epithelial cell death and proliferation in OSF and normal tissues.

## Materials and methods

### Cell lines and treatments

Primary human gingival fibroblast (hGF) cells (derived from Gingival biopsies) 11, and human keratinocytes (HaCaT) 12 were maintained in DMEM (Sigma-Aldrich, St. Louis, MO, USA) supplemented with 10% foetal bovine serum (FBS; Certified grade, Invitrogen Corporation, Grand Island, NY, USA, heat inactivated for HaCaT cells). Serum containing medium was supplemented with 100 units/ml penicillin and 100 μg/ml streptomycin (Invitrogen Life Sciences, Grand Island, NY, USA) and cells were grown at 37°C in a humidified chamber with 5% CO_2_. For treatments with various components, HaCaT and hGF cells were cultured in serum deprived medium (0.2% serum for hGF cells) for various time intervals and the components were added which include different alkaloids (Arecoline 400 μM, Arecaidine 1000 μM, Guvacine 1000 μM); Copper sulphate 50 μM; Iron Chloride. For treatment with Activin like kinase (ALK5) inhibitor (TβRI inhibitor, SB 431542; Sigma-Aldrich) or insulin-like growth factor (IGF)-1R inhibitor (407247; Calbiochem, MA, USA), cells were pre-treated with 10 μM of inhibitor for 2 hrs prior to the addition of the respective factors. For ROS neutralization cells were pre-treated with *N*-acetyl-l-cysteine (NAC; 1 mM) for 2 hrs before addition of different factors.

### Oral submucous fibrosis tissues

All the OSF and normal tissue samples used in this study were obtained from patients visiting D.A. Pandu Memorial R.V. Dental College, Bangalore, India. Tissue samples were collected after obtaining informed consent of patients with the approval of Institute Ethics Committee. Surgically excised tissues were preserved in RNA later (Ambion, Inc., St. Austin, TX, USA) at −80°C for RNA extraction. Part of the tissues was also fixed in buffered formalin for IHC. Normal tissues were obtained from non-OSF patients who underwent third molar extractions and without any associated inflammation. The tissue sections were examined by a pathologist to confirm the diagnosis of OSF using the following criteria:

The inclusion/exclusion criteria for the selection of patients are as follows.

#### Inclusion criteria

Restricted mouth opening less than 25 mm; hardened buccal mucosa on clinical examination; histopathological confirmation of incision biopsy.

#### Exclusion criteria

Submucous fibrosis associated with other premalignant conditions like Leukoplakia, Erythroplakia *etc*.; patients with immuno-compromised conditions; patients having chronic systemic disorders such as scleroderma, cystic fibrosis *etc*. and patients not willing for follow-up.

All OSF patients were regular chewers of areca nut in different preparations.

### RNA extraction and real-time RT-PCR

Total RNA was extracted from tissues and cells using TRI-reagent (Sigma-Aldrich) according to the manufacturer’s protocol. For cDNA synthesis, two micrograms of RNA was reverse transcribed using a cDNA synthesis kit (Applied Biosystems, Grand Island, NY, USA) and 1/100th of the reaction product was used per 20 μl PCR reactions. Real time PCR quantitations were performed in ABI Prism 7900HT sequence detection system and analysed with SDS 2.1 software (Applied Biosystems). The reactions were performed using Dynamo™ SYBERgreen 2× mix (Finnzymes, Keilaranta, Finland) in triplicate reactions. Expression of RPL-35A was used for normalization. The sequences of the primers used for PCR are listed in Table S1. The differential expression was determined by the formula

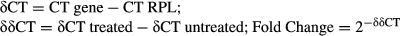


### Immunohistochemistry

Immunohistochemistry was performed according to previously published protocol 11. Before IHC all the tissue sections were routinely examined after haematoxylin and eosin staining. Briefly, tissues were deparaffinized in xylene for 15 min. and then transferred to absolute ethanol for 10 min. followed by incubation in PBS, pH 7.2 for 5 min. Antigen retrieval was done by boiling the deparaffinized sections in 10 mM citrate buffer (pH 6.0) using a microwave oven for 10 min. at 850 W. These sections were allowed to cool to room temperature and washed twice in PBS for 5 min. each. All the sections were further treated with 5% hydrogen peroxide in methanol to block the endogenous peroxidase activity, followed by a PBS wash. Skimmed milk powder (5%) was used to block the background staining for 1 hr. The sections were then incubated over night at 4°C with primary antibody. The primary antibody used in this study is, Ki-67 (1:30, M7240; Dako, Glostrup, Denmark). This was followed by incubation with supersensitive non-biotin horseradish peroxidase detection system (QD440-XAK; Biogenex, Fremont, CA, USA). Diaminobenzidine (DAB; Sigma-Aldrich) was used as a chromogenic substrate. For Ki-67 and terminal deoxynucleotidyl transferase dUTP Nick End Labeling (TUNEL) positivity, labelling index (LI) was calculated by counting hundred cells from three independent areas of germinal or *supra* basal or keratin layer, using the following formula:

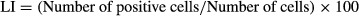


### MTT assay (cell cytotoxicity assay)

MTT (3-(4,5-dimethylthiazol-2-yl)-2,5-diphenyltetrazolium bromide) assay was performed as described previously 4. Briefly, 8000 cells were plated per well of 96-well tissue culture plates in 200 μl of culture medium supplemented with 10% FBS. The cells were allowed to attach for 24 hrs and treated with various factors [Arecoline, Arecaidine, Guvacine, transforming growth factor (TGF)-β, Areca nut *etc*.] for different time points in serum free medium or 0.2% serum containing medium for HaCaT and hGF cells, respectively. For inhibitor studies, the inhibitors (NAC - 1 mM, ALK5, and IGF-1R inhibitor −10 μM) were added 2 hrs prior to the addition of factors. Three hours before the stipulated time point, 20 μl of 5 mg/ml MTT (Sigma-Aldrich) solution was added to each well and was allowed to form formazan crystals. The culture medium was aspirated by vacuum suction and 200 μl of Dimethyl Sulfoxide (DMSO) was added in each well to dissolve the crystals. The intensity of the purple colour produced was estimated by determining the absorbance at 570 nm against DMSO blank using a spectrophotometer. Amount of formazan produced by the treated and untreated cells was compared and cell death was assessed by the decrease in % cell viability.

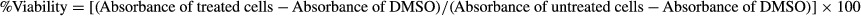


### Flow cytometric analysis

The DNA content of the cell is measured with the help of a flow Cytometer using propidium iodide as described previously 4. Briefly, 3 × 10^5^ HaCaT/hGF cells were plated per well of a 6-well tissue culture plate and allowed to grow for 24 hrs. The cells were then serum deprived for 24 hrs followed by the treatment with different concentrations of areca nut or arecoline for 48/72 hrs. The cells were trypsinized, collected in 1.5 ml centrifuge tubes, washed once with PBS and fixed by adding 900 μl of chilled 70% ethanol dropwise with constant shaking. The cell suspension was incubated at −20°C for 6 hrs, washed twice with PBS and were treated with 2 μg of DNAse-free RNAse for 12 hrs at 37°C to digest the cellular RNA. After the completion of digestion, 20 μl of 1 mg/ml propidium iodide solution was added into the mix and further incubated for 20 min. at 37°C. Flowcytometric analysis was performed by Fluorescence-activated cell sorting (FACS) Calibur (Becton and Dickinson, Franklin Lakes, NJ, USA) at FL1 channel and the distribution of cells in various cell cycle phases was quantitated (as % distribution) from the histogram using WinMDI.

### Detection of reactive oxygen species

Reactive oxygen species was quantitated by the DCFDA (2′,7′-dichlorodihydrofluorescein diacetate) staining 13. The DCFDA-AM (D6883; Sigma-Aldrich) is a membrane permeable molecule and upon stimulation by an ROS inducing agent, increase in fluorescence signal over the time is observed. For the detection of ROS generation by Flow Cytometric analysis, 2 × 10^5^ HaCaT/hGF cells were plated in each well of 6-well tissue culture plates and grown for 24 hrs. Cells were serum deprived for 24 hrs (0.2% FBS for hGF) and treated with various concentrations of areca nut extract or its fractions for 30 min. The cells were harvested by trypsinization and single cell suspension of cells was made in PBS. The cells were then treated with 1 μM DCFDA solution in DMSO in dark for 30 min. at room temperature. The distribution of DCFDA stained HaCaT/hGF cells was determined by flowcytometry in the FL-1 channel and their relative mean fluorescence intensities were plotted on Y axis.

### Catalase assay

Protocol for catalase assay was as prescribed by Sigma-Aldrich based on published reports 14,15. All the reagents were purchased from Sigma-Aldrich, USA. Briefly, 3 × 10^5^ HaCaT or hGF cells were cultured in 6-well tissue culture plates and were allowed to grow for 24 hrs to 70% confluency. The cells were serum starved for 24 hrs (0.2% for hGF) and then treated with areca nut extracts for 30 min. After the treatment, cells were washed once in chilled PBS and lysed in 200 μl of lysis buffer containing 50 mM Potassium phosphate buffer (pH 7.0 at 25°C), TritonX100 (1%) and 1% glycerol. The protein content of the lysates was estimated using Bradford method (Bio-Rad Protein Assay, Hercules, CA, USA). Equal amounts of total protein (30 μg) in the cell lysates were taken for catalase (EC 1.11.1.6) assay. In a 0.3 ml reaction mix, the final concentrations were 50 mM potassium phosphate (pH 7.0 at 25°C), 0.035% (w/w) H_2_O_2_ and 20 μl of cell lysate. Time taken in minutes (T) for the reduction of absorbance at 240 nm (ΔA240) from 0.45 to 0.40 of the assay mix was measured using a spectrophotometer (SmartSpec™ Plus; Bio-Rad). The enzyme activity was calculated using the formula and plotted on *Y*-axis;

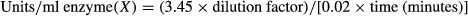


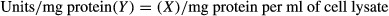


### DNA fragmentation assay

Inter-nucleosomal DNA fragmentation (200 base pairs) by the activated nucleases is a hallmark of apoptosis leading to formation of a ladder like pattern 16. To study apoptosis, HaCaT cells were grown in 60 mm tissue culture dishes and treated with areca nut cytotoxic (16 μg/ml) and sub-cytotoxic (4 μg/ml) concentrations for 72 hrs in the absence of serum. After removing the medium, cells were washed in Dulbecco’s Phosphate-Buffered Saline (DPBS) and were trypsinized using 1× trypsin followed by neutralization with reconstituted DMEM. Subsequently cells were lysed in 400 μl of lysis buffer containing 10 mM Tris-HCl (pH 8), 20 mM ethylenediaminetetraacetic acid (EDTA), and 0.2% Triton-X100 on ice for 20 min. Lysed cells were centrifuged at 12,000 × g for 20 min. to pellet cellular debris. The supernatant which contains genomic DNA was mixed with equal volume of phenol chloroform (1:1 ratio) and gently vortexed to remove the proteins. Following phase separation, genomic DNA in the upper aqueous phase was precipitated using 1/10th volume of sodium acetate (pH 5.2) and 2.5 volumes of absolute ethanol. The genomic DNA pellet was washed with 70% ethanol and was finally re-suspended in 30 μl of TE [Tris-EDTA (pH 8) + RNAse A (Sigma-Aldrich)], followed by incubation at 37°C for 2 hrs. The samples were then mixed with 6× DNA loading dye and electrophoresed on 1% agarose gel for 4 hrs at 100 V and stained with ethidium bromide.

### Annexin V assay

Early stage of apoptosis involves the acquisition of surface changes by dying cells, one of them being exposure of a phospholipid like phosphatidylserine (PS). Fluorescein isothiocyanate (Annexin V-FITC) is a fluorescent probe which binds to PS in the presence of calcium 17.

To identify areca nut induced cell death *via* apoptosis, Annexin V staining was performed using APOAF kit (Sigma-Aldrich). Briefly, 4 × 10^5^ HaCaT cells were plated in each of a 6-well tissue culture plate. Cells were treated with areca nut water (ANW) and ethanol extracts (ANE) for 36 hrs followed by trypsinization. Cells were washed twice in PBS and re-suspended in binding buffer at a density of 1 × 10^6^ per ml. All the samples were incubated with 5 μl of annexin V-FITC and 10 μl of propidium iodide, except for control cells where both PI and annexin was not added. These samples were subjected to flow cytometry.

### TUNEL assay

DNA fragmentation is a characteristic hallmark of apoptosis and TUNEL can be used to detect DNA fragmentation *in situ* by labelling the terminal end of nucleic acids 18.

For TUNEL assay, OSF and normal tissue sections were deparaffinized in xylene for 15 min. and then transferred to absolute alcohol for 10 min. followed by incubation in 1× PBS for 10 min. Tissue samples were incubated with 50 μl of Proteinase K solution at 37°C for 30 min. followed by two washes in deionized water. All the sections were further treated with 5% hydrogen peroxide (30%) in methanol to block the endogenous peroxidase activity, followed by a 1× PBS wash for 1 min. These samples were immersed in 1× TdT labelling buffer for 5 min. and then incubated with 50 μl of labelling reaction (containing TdT dNTP, TdT Enzyme, 1× Manganese Cation and 1× TdT labelling buffer) for 1 hr at 37°C. After the incubation, reaction was stopped using 1× TdT stop buffer for 5 min. Samples were washed twice in deionized water for 5 min. each and incubated with 50 μl of Strep-Horseradish peroxidase (HRP) solution (Secondary) for 10 min. at 37°C. After secondary incubation, samples were again washed twice in 1× PBS for 2 min. each. Finally, colorimetric substrate DAB along with enhancer and H_2_O_2_ was added for colour development. Sections were counter stained using haematoxylin and were later mounted using D.P.X Mountant.

### Preparation of Areca nut extracts and fractionation

Areca nut extract preparation and fractionation were performed according to previously described methods 6,19,20. Briefly, thirty grams of dried and de-husked Betel nut was ground and extracted by 100 ml of de-ionized water for 4 hrs at 4°C with constant stirring. Insoluble components were further extracted with ethanol. All the extracts were filtered, lyophilized and stored at 4°C. For treatments, the weighed dry powder was dissolved in de-ionized water and stored at −70°C.

Filtered water extract samples were partitioned with dichloromethane (DCM) in the ratio of 1:1 by volume. Then the water phase was collected and the impurities associated with DCM were also collected (DCM phase). The partitioning with DCM was repeated for three times and the water phase was further partitioned with ethyl acetate (1:1 by volume), which was repeated for three times. The ethyl acetate extracts were collected and evaporated to dryness with vacuum rotary evaporator. All the three phases namely dichloromethane (DCM phase), water (Alkaloid phase) and ethyl acetate (Polyphenol phase) were used for treating cells. The purity of alkaloid and polyphenol fractions of areca nut was established by Liquid chromatography-mass spectrometry (LC-MS) profiling as described in our previous report 6. The fractions thus obtained were found to be pure and did not show cross-contamination.

### DNA cleavage experiment

DNA cleavage experiment was performed using pUC19 plasmid (150 ng, 2686 base-pairs) by incubating it along with different combinations of arecoline (100 μg/ml), copper (150 μM), glutathione (Glut 5 μM), Superoxide dismutase (SOD 300 units), Sodium azide (NaN_3_), and DMSO (20 mM) in 50 mM Tris-HCl/NaCl buffer (pH = 7.2) for 24 hrs at 37°C. DNA plasmid cleavage products were analysed by gel electrophoresis (1% agarose gel) as reported earlier 21.

### Cyclic voltammetry

Cyclic voltammetric measurements were performed at 25°C on an EG&G PAR Model 253 VersaStat potentiostat/galvanostat (Scribner Associates Inc., Southern Pines, NC, USA) with electrochemical analysis software 270 using a three electrode set-up comprising a glassy carbon working, a platinum wire auxiliary and a saturated calomel reference electrode. Potassium chloride (0.1 M) was used as a supporting electrolyte in water. The electrochemical data were uncorrected for junction potentials.

### UV-Visible spectrometer analysis

To study the complex formation, 1:1 molar ratio of Cu(II)SO_4_ to arecoline was incubated for 24 hrs at 37°C in 50 mM Tris-HCl/NaCl buffer (pH = 7.2) and electronic absorption spectra were recorded using CuSO_4_ as reference.

### Mass spectrometry

Copper arecoline complex (1:1 molar ratio) mass spectra was recorded on Ultraflex MALDI TOF/TOF mass spectrometer (Bruker Daltonics, GmbH, Bremen, Germany) in the reflection mode using a 90 nsec. time delay, acceleration voltage was 25-kV in the positive ion mode. The system utilizes a 50 Hz pulsed nitrogen laser, emiting at 337 nm and α-Cyano-4-hydroxy cinnamic acid was used as a matrix.

### BrdU assay

5000 hGF cells were seeded in 96 well plates in triplicate in 200 μl media. Cells were serum deprived for 24 hrs in 0.2% serum containing medium. Cells were treated with different concentrations of ANW extract for 48 hrs. After 24 hrs treatment with areca nut, 10 μM bromodeoxyuridine (BrdU) was added (20 μl) and incubated for 24 hrs, coinciding with the end point of 48 hrs of areca nut treatment. Following this, media was removed, washed thrice with PBS and fixed with 0.1 N NaOH in 70% ethanol for 30 min at room temperature. Fixative was removed, washed twice with wash buffer and 100 μl of primary anti BrdU antibody (1:500) was added. Following incubation for 2 hrs at room temperature, cells were washed thrice and 100 μl secondary antibody (1:1000; anti-mouse HRP) was added and incubated for 1 hr at room temperature. Cells were washed twice with buffer and 100 μl TMB solution was added in dark and monitored for colour development (<10 min). Stop solution (0.2 N H_2_SO_4_) 100 μl was added immediately and reading was taken at dual wavelength of 450/595 nm. Background correction by reading of wells without cells but otherwise processed in same manner was performed along with negative control which has cells without BrdU.

### Western blot analysis

Proteins were extracted from cells after DPBS wash, using lysis buffer (Tris-Hcl 50 mM, NaCl 150 mM, SDS 0.1%, NP-40 0.5%, containing protease inhibitor cocktail, Sigma-Aldrich). Equal amount of protein (determined by Bradford method) extracted from cells were resolved on 12% SDS-PAGE gel, transferred to polyvinylidene difluoride membrane and subjected to immunoblot analysis. To block non-specific binding sites, blots were incubated in 5% non-fat dairy milk for 1 hr followed by overnight incubation in primary antibodies at 4°C, diluted according to the manufacturer’s instructions [pIGF-1R antibody, 1:1000, ab5681; Abcam (Cambridge, MA, USA), secondary antibody anti-mouse (1:2000); IGF-1R Antibody, 1:1000, [Cell Signaling Technology (CST),Danvers, MA, USA] #3027, secondary antibody anti-rabbit, 1:2000]. Following this horseradish peroxidase-conjugated secondary antibody (Anti- rabbit/mouse HRP conjugated secondary antibody Sigma-Aldrich, dilution 1:2000) was added and incubated for 1 hr. Proteins were visualized with a chemiluminescence detection system (Super Signal West Femto Chemiluminescent Substrate, 34095; Thermo Fisher Scientific Inc., Waltham, MA, USA) and subsequent exposure to X-ray film.

### Statistical analysis

Statistical analysis was performed using one-way anova among different treatments. All treatments were compared to untreated and the observed significance levels from multiple comparisons were made using the Bonferroni’s Multiple Comparison Test and *P* ≤ 0.05 was considered as significant. *P*-value ≤0.01, ≤0.001 and ≤0.0001 are indicated by *, **, and *** respectively.

## Results

### Differential effect of areca nut extracts on epithelial and fibroblast cells

**Figure 1:**
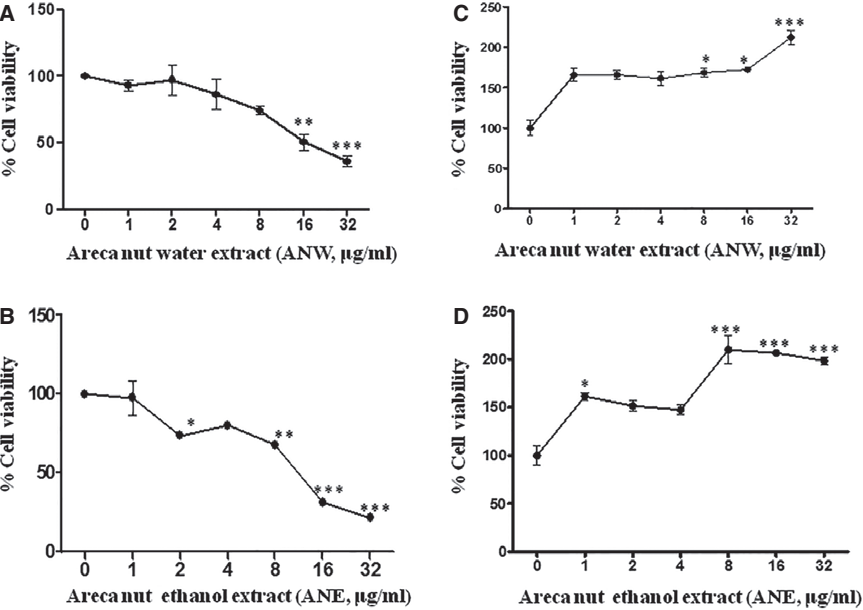
Effect of areca nut extracts on keratinocyte and fibroblast cells. Eight thousand HaCaT cells (keratinocytes) were plated in 96 well tissue culture plates and treated with 0–32 μg/ml of areca nut (A) water (ANW) and (B) ethanol (ANE) extracts for 48 hrs under serum free conditions as described in materials and methods. Similarly hGF cells (fibroblasts) were also treated with (C) ANW and (D) ANE extract but in 0.2% serum containing medium and MTT assays were performed as described in methods. The results are shown as % viability with respect to untreated cells (representing 100%) and each dot represent mean ± SEM of biological triplicate readings. Statistical significance was determined using one-way anova among different treatments using the Bonferroni’s Multiple Comparison Test with *P* ≤ 0.05 indicating significance (*P*-value ≤0.01, ≤0.001 and ≤0.0001 are indicated by *, **, and *** respectively). % Cell viability = [(Absorbance of treated cells − Absorbance of DMSO)/(Absorbance of untreated cells − Absorbance of DMSO)] × 100.

**Figure 2:**
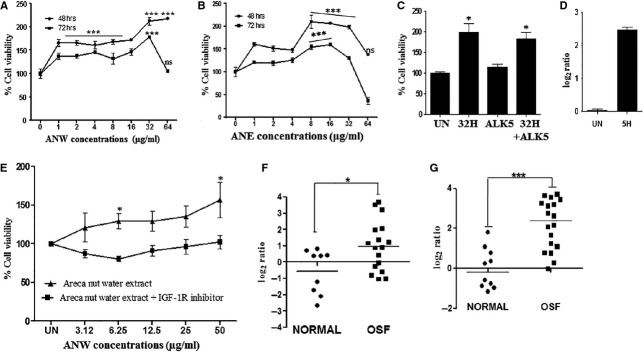
Areca nut induced proliferation of hGF cells is dependent on IGF signalling. Eight thousand human gingival fibroblast cells (hGF) were plated in 96 wells tissue culture plates and treated with 0–64 μg/ml of (A) ANW and (B) ANE for 48 and 72 hrs in 0.2% serum containing medium. (C) ALK5 (TGF-β RI inhibitor) has no effect on areca nut induced proliferation of hGF cells highlighting no involvement of TGF-beta signalling. (D) Areca nut induces expression of IGF-II in hGF cells. The bars depict q-PCR quantitation performed in triplicates as described in methods. (E) IGF-1R inhibitor treatment (for 1 hr) affects areca nut induced proliferation of hGF cells as shown by % viability (sub-cytotoxic concentration of IGF-1R inhibitor was used with % viability of 89.65%). OSF tissues show higher expression of IGF-1 (F) and IGF-II (G) compared to normal tissues as determined by q-PCR. Log2 transformed fold change is plotted for each sample in the scatter plot. Statistical significance was determined as described in materials and methods (5H: 32H - 5 and 32 μg/ml of ANW; UN: Untreated; ALK5: ALK5 inhibitor, 32H+ALK5- Areca nut water extract (32 μg/ml) +ALK5 inhibitor).

In order to study the effects of areca nut extracts on epithelial and fibroblast cells, they were treated with increasing concentrations of ANW and ANE. Both extracts showed cytotoxicity on keratinocytes (HaCaT) with IC_50_ of 16 and 9.5 μg/ml by ANW and ANE, respectively (Fig. 1A and B) suggesting higher cytotoxicity of ethanol extract. In contrast, ANW and ANE were pro-proliferative on hGF cells (hGF). As shown in Figure 1C and D, treatment of hGF cells with as low as 1 μg/ml of ANW and ANE resulted in a 60% increase in the proliferation of hGF cells. Interestingly, there was a biphasic response to both these extracts suggesting involvement of more than one component in this response. We also confirmed proliferation of hGF cells by areca nut extracts using BrdU assay which shows increased incorporation of BrdU in areca nut treated hGF cells (Fig. S1A). Exposure of hGF cells to either higher concentrations of areca nut extracts (>32 μg/ml) or for longer durations (72 hrs) led to the compromise of induced proliferation or resulted in the inhibition of growth (Fig. 2A and B).

### Areca nut induced proliferation of fibroblast cells is IGFR dependent

To understand the pro-proliferative actions of areca nut extracts on hGF cells, we treated hGF cells with areca nut extracts in the presence of TβRI inhibitor (TGF-β Receptor I inhibitor/ALK5 inhibitor), as TGF-β is known to promote fibroblast proliferation 22. However, areca nut induced proliferation of fibroblast cells was not compromised in the presence of ALK5 inhibitor (Fig. 2C). This observation is also in line with our earlier results where, there was no apparent induction of TGF-β pathway by areca nut extracts in hGF cells 6. Insulin-like growth factor pathway is known for its pro-proliferative role in fibroblast cells and has been shown to be regulated by arecoline 23. Hence, we studied the regulation of IGFs by areca nut extracts in hGF cells. As shown in Figure 2D, IGF-II is induced by areca nut extract to 2.5-fold over controls while IGF1 basal expression was found to be extremely low in hGF cells. Furthermore, we studied the activation of IGF pathway by the phosphorylation of IGF-1R. It was observed that pIGF-1R levels were induced by ANW with no effect on total IGF-1R levels (Fig. S1B). To establish the areca nut induced growth promotion through IGF signalling pathway, we treated hGF cells with ANW extract in presence of IGF-1R inhibitor and expectedly, areca nut induced proliferation was compromised (Fig. 2E). In good correlation, both IGF-1 and IGF-II were found to be significantly up regulated in OSF tissues, suggesting a possible role for IGF signalling in the OSF manifestation (Fig. 2F and G).

### Differential induction of ROS in HaCaT and hGF cells by areca nut and its role in HaCaT cytotoxicity

**Figure 3:**
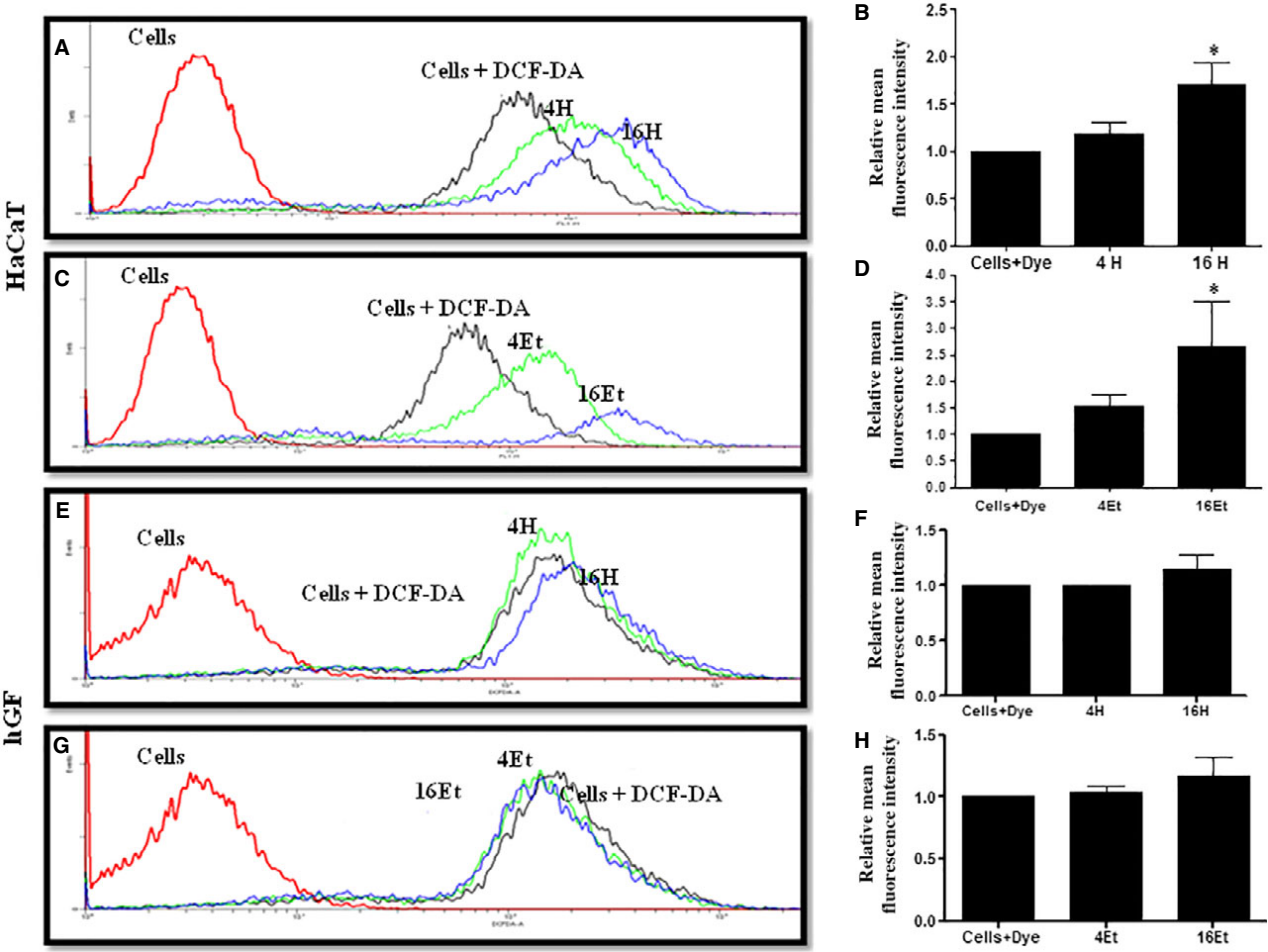
Areca nut extracts induce ROS in HaCaT cells but not in hGF cells. HaCaT and hGF cells were treated with ANW (**A, B, E** and **F**) and ANE extracts (**C, D, G** and **H**) for 30 min. followed by incubation with 1 μM DCF/DA for 20 min. in dark. A flowcytometric analysis was performed and distribution of differentially stained cells was determined. B, D, F and H are quantitations of induced ROS in HaCaT cells and hGF cells by Area nut extracts, respectively (Red: Cells alone; Black: Cells + DCF/DA; 4H: 16H- ANW 4 and 16 μg/ml; 4Et: 16Et- ANE 4 and 16 μg/ml).

Cytotoxicity by ROS is a well-known phenomenon 24. To understand the possible mechanism of areca nut cytotoxicity on HaCaT cells, ROS generation was measured as some of the areca nut components are known to induce ROS 25. Both ANW and ANE extracts induced ROS in HaCaT cells in a dose dependent manner when treated for 30 min. in serum free conditions (Fig. 3A–D). Also, ANE induced more ROS compared to ANW in HaCaT cells. In contrast, upon exposure of areca nut extracts (ANW and ANE) on hGF cells, there was no significant increase in ROS (Fig. 3E–H). Therefore, areca nut induced cytotoxicity of HaCaT cells could be mediated by ROS.

**Figure 4:**
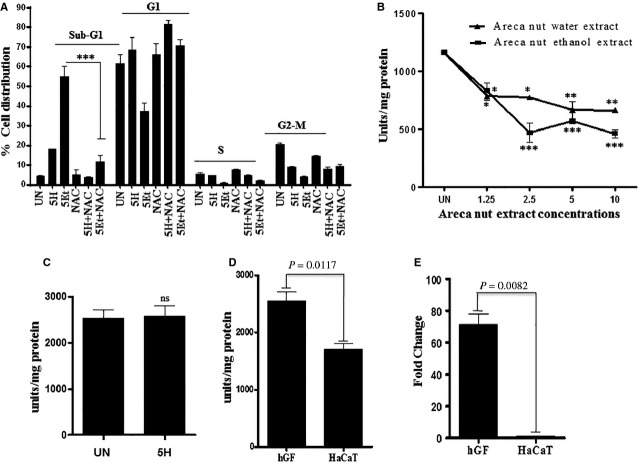
Areca nut induced cytotoxicity on HaCaT cells is ROS mediated. HaCaT cells were treated with ANW, ANE and/or with *N*-acetyl-l-cysteine for 48 hrs and cell death was analysed based on Sub-G1 peak of flowcytometry analysis. (A) Quantitation of flowcytometric analysis showing distribution of cells in each phase of cell cycle upon various treatments. (B) HaCaT cells were treated with increasing concentrations of areca nut extracts for 30 min. Equal amount of protein was assayed for catalase activity (units/mg protein) as described in methods. (C) hGF cells were treated with ANW (5H, 5 μg/ml) for 30 min. and catalase activity was estimated. (D and E) Bars show relative catalase activity and HOX-1 expression in hGF and HaCaT cells, respectively. Statistical significance was determined using one-way anova (*P*-value ≤0.01, ≤0.001 and ≤0.0001 are indicated by *, **, and ***, respectively).

To test the possibility that areca nut induced ROS in HaCaT cells is responsible for its cytotoxicity, we treated HaCaT cells with ANW and ANE in the presence of NAC (a cell permeable glutathione synthesis precursor) and monitored cell death by FACS analysis. Both ANW and ANE failed to induce cell death in the presence of NAC, as highlighted by the decrease in Sub-G1 population (Fig. 4A). This confirmed that areca nut induced cytotoxicity of HaCaT cells is mediated by ROS.

### Areca nut induced ROS in HaCaT cells is due to suppression of catalase activity

Reactive oxygen species levels are maintained by the balance between its generation and degradation. There are several enzymes involved in ROS generation, one of the prominent being NADPH oxidase while ROS neutralization is mainly by superoxide dismutase and catalase. A previous report from our laboratory has shown arecoline’s suppressive actions on catalase activity in keratinocytes 4. Therefore, we hypothesized that similar to arecoline, areca nut may induce ROS in HaCaT cells by suppressing catalase activity. To test this, we treated HaCaT cells with increasing concentrations of areca nut extracts and measured catalase activity in the cell lysates. Both ANW and ANE suppressed catalase activity in a dose dependent manner in HaCaT cells (Fig. 4B). Also ANE extract suppressed catalase activity more than ANW which correlates with higher ROS generation by the ANE in HaCaT cells. But there was no suppression of catalase activity by ANW in hGF cells (Fig. 4C) which also correlates well with the lack of ROS generation in hGF cells.

To understand the selective ROS generation by areca nut extracts in HaCaT but not in hGF cells, we compared basal catalase activity of HaCaT and hGF cells. Basal catalase activity in hGF cells was higher (1.5 times) compared to HaCaT cells; highlighting the higher ROS neutralization potential of hGF cells (Fig. 4D). To further understand the ROS neutralization potential of both the cell types, we compared basal expression of Heme oxygenase-1, an anti-oxidant enzyme known for its cytoprotective role 26. Heme oxygenase-1 expression in hGF cells was much higher than in the HaCaT cells (Fig. 4E), further strengthening the observation that hGF cells have higher ROS neutralization potential compared to HaCaT cells. Therefore, it is possible that lack of ROS generation by areca nut in hGF cells could also be because of their higher ROS neutralization potential.

### Alkaloids of ANW are responsible for areca nut cytotoxicity in HaCaT cells

**Figure 5:**
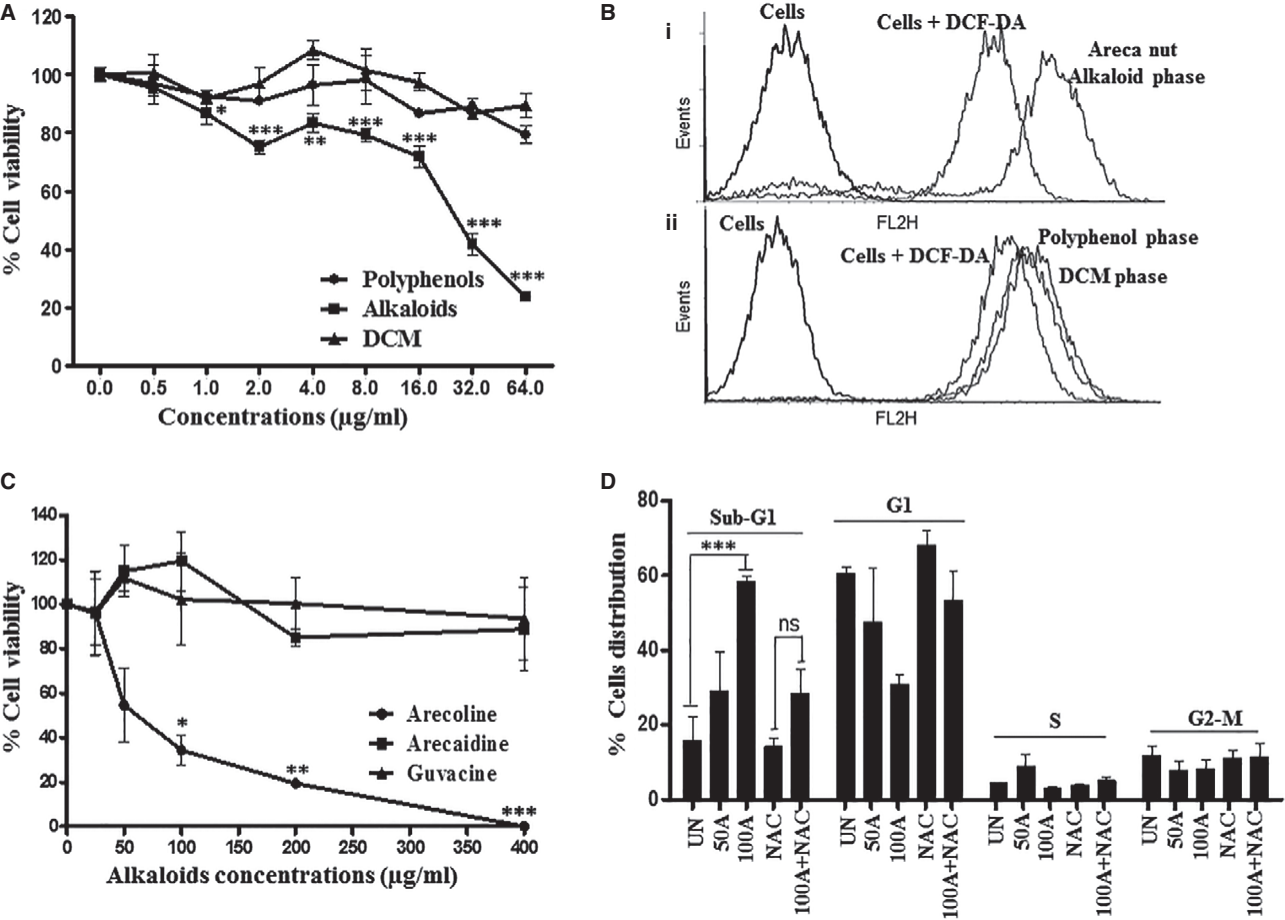
Areca nut induced cytotoxicity on HaCaT cells is mediated by arecoline. (A) Eight thousand HaCaT cells were plated in 96 wells tissue culture plates and treated with 0–64 μg/ml of different areca nut fractions (Alkaloid, Polyphenol and DCM) for 48 hrs under serum free conditions. MTT assay was performed as described in materials and methods and % viability is plotted. (B) HaCaT cells were treated with areca nut alkaloid (i), areca nut polyphenol and DCM phases (ii) for 30 min and then incubated with 1 μM DCF/DA for 20 min. in dark. A flowcytometric analysis was performed and distribution of differentially stained cells was determined. (C) MTT assay was performed on HaCaT cells treated with different alkaloids for 48 hrs. (D) HaCaT cells were treated with arecoline with or without *N*-acetyl-l-cysteine for 48 hrs and cell death was analysed using Sub-G1 peak of flowcytometry. Graph represents the quantitation of biological triplicate experiments.

To find out areca nut component responsible for its cytotoxicity on HaCaT cells, we fractionated ANW into Water (Alkaloid), Ethyl acetate (Polyphenol) and DCM (Impurity) phases as described in the materials and methods. Treatment of HaCaT cells with Alkaloid phase was found to be cytotoxic while Polyphenol and DCM phases were not able to induce cytotoxicity (Fig. 5A). This is also in line with the higher ROS generation potential of alkaloid phase compared to polyphenol and DCM phases as shown by the DCF-DA assay (Fig. 5B, i and ii). Therefore, alkaloids in areca nut mediate cytotoxicity of HaCaT cells.

Constituents of areca nut alkaloid phase are well-known in the literature 20. Therefore, to further explore the areca nut component in alkaloids responsible for inducing cytotoxicity in HaCaT cells, we treated these cells with increasing concentrations of different alkaloids of areca nut namely arecoline, arecaidine and guvacine. Among all the alkaloids treated on HaCaT cells, only arecoline was found to be cytotoxic to HaCaT cells with an IC_50_ of 55 μg/ml (IC_50_ of ANW is 16 μg/ml) (Fig. 5C). Therefore, areca nut cytotoxicity on HaCaT cells was predominantly due to alkaloids and arecoline is the principal alkaloid responsible for this cytotoxicity.

### Arecoline induced cytotoxicity in HaCaT cells is ROS dependent

The induction of ROS by arecoline in HaCaT cells has been reported previously 4. If arecoline is the mediator of areca nut cytotoxicity, arecoline mediated cell death would also be compromised in the presence of NAC. Expectedly, arecoline failed to induce cytotoxicity in presence of NAC, as highlighted by the reduction in sub-G1 peak (Fig. 5D). Therefore, arecoline induced cell death in HaCaT cells is also ROS mediated.

### Areca nut and arecoline induced cell death is due to apoptosis in HaCaT cells

**Figure 6:**
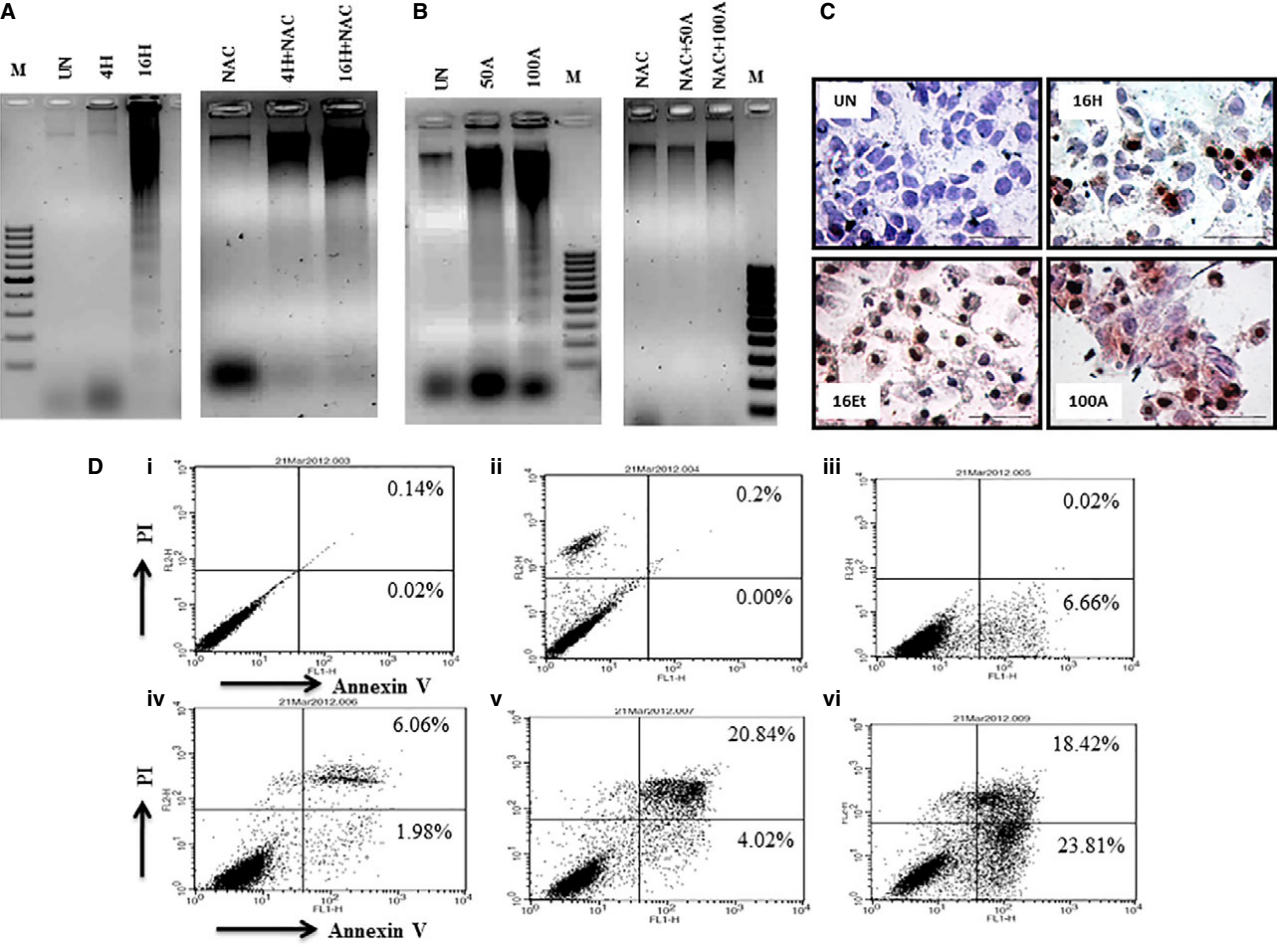
Areca nut and arecoline induce apoptosis in HaCaT cells. HaCaT cells were treated with (A) ANW or (B) arecoline with/without *N*-acetyl-l-cysteine for 48 hrs and cell death was analysed using DNA ladder assay as described in the materials and methods. Genomic DNA from HaCaT cells was resolved on 1.5% agarose gel containing ethidium bromide. (C and D) Cell death induced by areca nut and arecoline on HaCaT cells was also visualized by TUNEL and Annexin V positivity [M: 100 bp ladder; UN: Untreated; 4H or 16H: ANW (4/16 μg/ml); 16Et: ANE (16 μg/ml); NAC: *N*-acetyl-l-cysteine (NAC-1 mM); 50A/100A: Arecoline (50/100 μg/ml); i: Cells alone; ii: PI alone; iii: Annexin alone; iv: Annexin + PI (untreated); v: Arecoline (100 μg/ml); vi: ANW (16 μg/ml)].

As demonstrated, arecoline could be the mediator of areca nut induced cytotoxicity in HaCaT cells. Hence, the nature of cell death induced by areca nut and arecoline must be similar. To elucidate the nature of cell death induced by areca nut and arecoline in HaCaT cells, we performed DNA fragmentation assay, TUNEL assay and Annexin V staining, which are indicators of apoptosis. Upon addition of 16 μg/ml of areca nut extract, DNA ladder formation was observed which was compromised in the presence of NAC (Fig. 6A). Similarly, treatment of HaCaT cells with 50 μg/ml arecoline induced DNA ladder formation, which was compromised in the presence of NAC (Fig. 6B). Furthermore, treatment of cytotoxic concentration of both areca nut and arecoline induced TUNEL positivity in the nuclei (Fig. 6C) of HaCaT cells. Similarly, Annexin V staining showed that arecoline induced cell death has higher late apoptotic positivity (20.84%) compared to early apoptosis (4.02%) while areca nut induced cell death has both high positivity for early (23.81%) and late (18.42%) apoptosis (Fig. 6D, i–vi). Therefore, the above results demonstrate that both areca nut and arecoline induced cell death in HaCaT cells by inducing apoptosis in an ROS dependent manner.

### Arecoline and copper synergize to induce HaCaT cytotoxicity in an ROS dependent manner

**Figure 7:**
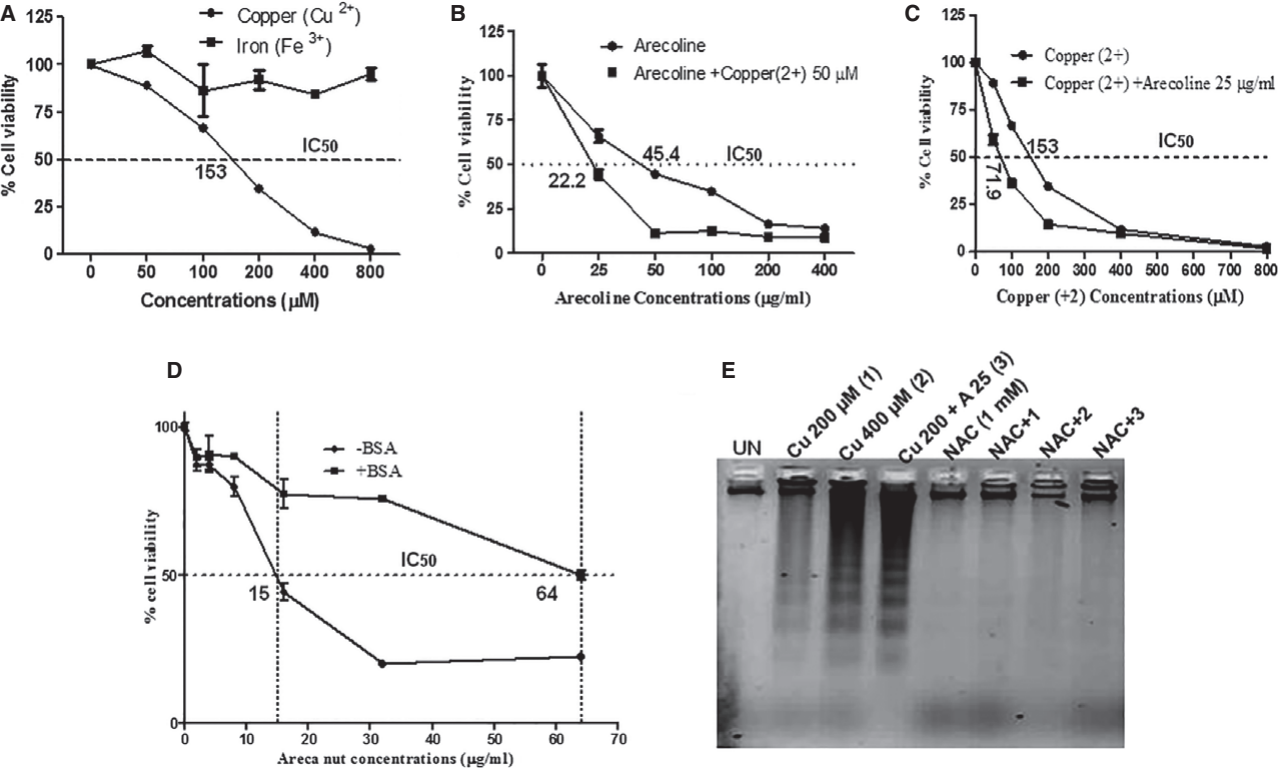
Arecoline and copper induces HaCaT cytotoxicity in an ROS dependent manner. MTT assay was performed on HaCaT cells treated with (A) Fe (3+), Cu (2+); (B) Arecoline with or without Cu (2+); (C) Cu (2+) with or without arecoline; (D) ANW with or without BSA (0.1%) in serum free medium for 48 hrs. (E) HaCaT cells were treated with copper with or without arecoline for 48 hrs and DNA fragmentation was analysed using DNA ladder assay as described in the materials and methods. Genomic DNA was resolved on 1.5% agarose gel containing ethidium bromide. The DNA fragmentation was neutralized by *N*-acetyl-l-cysteine.

Our results suggest that arecoline could be the mediator of areca nut cytotoxicity but the IC_50_ of ANW (16 μg/ml) is much lower than the IC_50_ of arecoline (55 μg/ml), suggesting the involvement of other component(s) of areca nut in mediating areca nut cytotoxicity. Areca nut is known to contain metals including copper and iron that could contribute to its cytotoxicity. We tested both iron and copper and only copper (Cu^2+^) was found to be cytotoxic on HaCaT cells (Fig. 7A). As ANW possess both arecoline and copper, we treated HaCaT cells with varying concentrations of arecoline in the presence of sub cytotoxic concentration of copper (50 μM) and assessed the cytotoxicity. As shown in Figure 7B, copper potentiated arecoline cytotoxicity and shifts its IC_50_ to 22.2 μg/ml from 45.4 μg/ml (Fig. 7B). This IC_50_ is closer to IC_50_ of ANW on HaCaT cells. Similar potentiation of cytotoxicity was also observed with varying concentrations of copper in presence of arecoline (Fig. 7C), highlighting the possible role of copper along with arecoline in mediating areca nut cytotoxicity. As areca nut composition shows presence of several metals, we used bovine serum albumin (BSA), a general quencher of metals, which has a metal binding domain 27, to neutralize the contribution of metals in areca nut cytotoxicity. Presence of BSA reduced the ANW cytotoxicity and shifts its IC_50_ closer to arecoline alone (64 μg/ml; Fig. 7D). In addition, the potentiation of arecoline’s cytotoxicity by copper also resulted in concomitant increase in apoptosis (DNA ladder) in an ROS dependent manner (Fig. 7E). These results unequivocally establish that the cytotoxicity of areca nut is contributed by alkaloids along with metals such as copper.

### Arecoline potentiates oxidation reduction potential of copper leading to increased DNA cleavage in superoxide (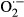) dependent manner

**Figure 8:**
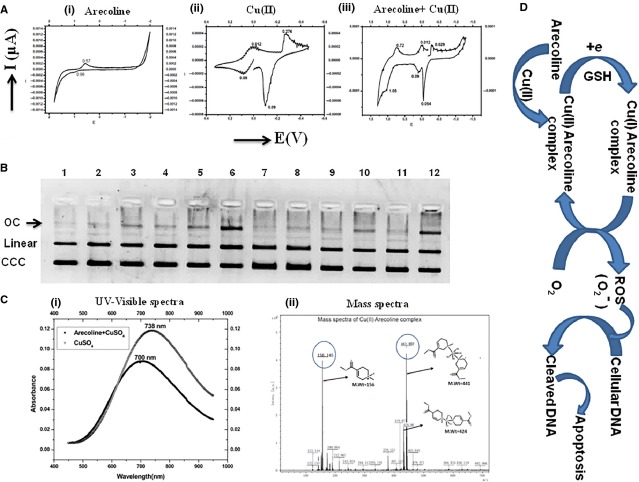
Arecoline potentiates oxidation reduction potential of Cu (II). Cyclic voltammograms of arecoline, Cu(II) and both. Arecoline potentiates oxidation reduction potential of copper as shown by the cyclic voltammograms. (B) Plasmid cleavage assay showing increased cleavage of plasmid (Nicked/Open circular) by arecoline and Cu (II) in presence of glutathione, which gets neutralized in presence of SOD (highlighted by an arrow). (C, i) UV-Visible Spectra of CuSO_4_ and CuSO_4_^+^ Arecoline showing shift in absorbance peak. (C, ii) Mass spectra of arecoline and CuSO_4_ incubated together showed a characteristic peak corresponding to [Cu(Arecoline)_2_(OH)_4_] complex at 441.057 with grater abundance, however, fragmented peak of complex [Cu(Arecoline)_2_(OH)_3_] was observed at 424. (D) Schematic diagram showing possible mechanism of synergistic cytotoxicity of arecoline and Cu (II). (OC: open circular; CCC: covalently closed circular forms of plasmid; 1: DNA; 2: DNA+A100; 3: DNA+ Cu150; 4: DNA+ Glut; 5: DNA+A100+Cu150; 6: DNA+A100+Cu150+Glut; 7: DNA+SOD300; 8: DNA+A100+Cu150+Glut+SOD300; 9: DNA+NaN_3_ 20; 10: DNA+A100+Cu150+Glut+NaN_3_; 11: DNA+ DMSO20; 12: DNA+A100+Cu150+Glut+DMSO20).

In an attempt to understand increased cytotoxicity of arecoline in presence of copper, cyclic voltammetry studies were performed. Cyclic voltammetry studies revealed presence of oxidation-reduction peaks of arecoline and copper (II), indicating transfer of electrons when arecoline and copper (II) are combined (Fig. 8A). We also performed plasmid cleavage assay which suggests an increased potential of copper to cleave DNA with arecoline in the presence of glutathione (Fig. 8B). This increased plasmid cleavage capacity was completely neutralized upon addition of SOD (Superoxide dismutase) while partially rescued by sodium azide (Quencher of singlet oxygen; O_2_^−^) but does not get neutralized with DMSO (Scavenger of hydroxyl radical; Fig. 8B). These results suggest a possible role of superoxide radical in arecoline and copper mediated synergistic cytotoxicity. Moreover, addition of glutathione (an abundant cellular tripeptide) increased the cleavage of the plasmid due to increased ROS generation. Furthermore, to see the direct interaction between arecoline and copper, DNA UV spectrophotometric studies were performed. UV-Visible Spectra of CuSO_4_ shows characteristic absorption at 738 nm. However, when CuSO_4_ was incubated with arecoline, it showed absorption peak at 700 nm (Fig. 8C, i). Cupric complexes show d-d transition in the range 600–700 nm. A shift in d-d transition absorption of CuSO_4_ in the presence of arecoline possibly indicates complex formation, which is further supported by mass spectrometry studies (ESI-MS). Mass spectra of arecoline and CuSO_4_ incubated together showed a characteristic peak corresponding to [Cu(Arecoline)_2_(OH)_4_] complex at 441.057 with greater abundance, however, fragmented peak of complex [Cu(Arecoline)_2_(OH)_3_] was observed at 424 (Fig. 8C, ii). Peak corresponding to arecoline was observed at 156. The above ESI-MS result confirms complex formation between arecoline with copper.

Therefore in light of the above results, we propose that when copper (II) is combined with arecoline, it leads to the formation of arecoline copper complex in which arecoline gets oxidized resulting in reduction of copper (II) to Cu (I). Reduced Cu (I) in turn donates an electron to O_2_ leading to the formation of superoxide radical. This superoxide radical could possibly be responsible for DNA cleavage (damage) which could generate an apoptotic response in the cell (Fig. 8D).

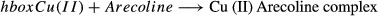


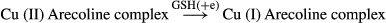


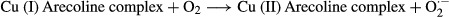


### Evidence of apoptotic cells in the epithelium of OSF tissues by TUNEL staining

**Figure 9:**
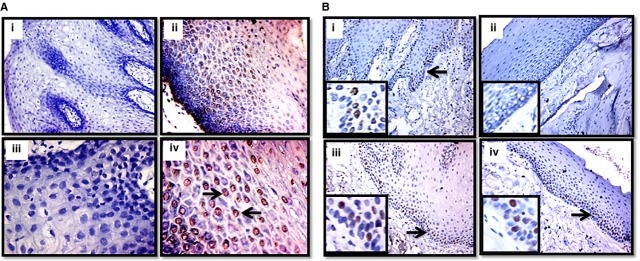
Atrophied epithelium of OSF has higher apoptotic index and mixed Ki-67 cells positivity. TUNEL assay was performed on normal (A, i and iii) and OSF tissue (A, ii and iv) sections. TUNEL positive nuclei can be seen in the epithelium of OSF tissues compared to no staining in normal tissues as indicated by arrows (i and ii are 20× images and iii and iv are 40× images). (B) Representative images of immunohistochemistry in normals (B, i and iii) and OSF (B, ii and iv) tissues stained with Ki-67 antibody (brown nuclei, arrows). The above images show Ki-67 negative (B, ii) and high Ki-67 (B, iv) labelling index in the epithelium of OSF tissues, compared to normals where all the tissues were moderately positive for Ki-67 (B, i and iii) B, i–iv are 20× images and their inset shows 40× magnification.

Oral epithelium of betel nut chewers or OSF patients is constantly exposed to areca nut and in light of our data, could be dying of apoptosis. To explore this, we performed TUNEL assay on OSF and normal tissues as described in the methods. The data revealed a higher percentage of OSF epithelial cells showing TUNEL positivity (LI = 0–60%) compared to epithelial cells of normal tissues (LI = 0–2%; Fig. 9A and Table S2). Also, 31.25% of the OSF patients had higher LI (≥25%) and an equal percentage of OSF patients had moderate TUNEL positivity (LI = 2–24%) while 37.5% patients were TUNEL negative (LI = 0–1%). TUNEL positivity was predominantly seen in the upper keratin and *supra* basal layer of the epithelium. In all the tissues (both OSF and normal), the basal epithelial cells were found to be unaffected. Taken together, apoptosis of epithelial cells could be one of the reasons for epithelial atrophy seen in OSF.

### Atrophied epithelium of OSF patients is positive for Ki-67

Based on the above results, we hypothesized that the proliferation of OSF epithelium might also be affected. To address this, we performed Ki-67 immuno-staining (a proliferation marker) on OSF and normal tissues. We observed that all the normal epithelial cells showed nuclear Ki-67 positivity (LI = 3–17%) in the germinal layer of epithelium and 29.4% of the OSF patients showed higher staining for Ki-67 (LI ≥25%), 35.2% showed moderate staining (LI <25 ≥ 2%) while 35.2% of OSF patients were negative (LI: 0–1%) for Ki-67 staining (Fig. 9B and Table S2). Labelling index of Ki-67 ranged from 3% to 17% in normal tissues while it was 0–58% in OSF tissues. Careful analysis of TUNEL positive and Ki-67 positive sections revealed that OSF patients with high TUNEL positivity had very high Ki-67 LI (≥25%). But the positivity for Ki-67 is in the basal layer while TUNEL positivity lies in the *supra* basal or keratin layer of the epithelium.

## Discussion

Habitual chewing of areca nut in various formulations has been proposed as a principal etiological factor to develop several oral lesions including OSF, oral cancer *etc*. Significant changes in the oral epithelial (atrophy) and fibroblast cells (fibrosis) of OSF patients have been reported 28. These changes have been attributed to areca nut chewing habit. Therefore, studies on the response of keratinocytes and fibroblasts to areca nut extracts could give better insights into the OSF pathogenesis. In this study, we employed *in vitro* studies to elucidate the effects of areca nut extracts and validated some of these results in the OSF tissues.

### Areca nut promotes proliferation of fibroblasts

Our study revealed pro-proliferative effect of areca nut extracts on fibroblast cells. This was found to be limited to lower concentrations of areca nut and for a shorter duration. Areca nut extract induced proliferation of fibroblasts (hGF) was found to be dependent on IGF signalling. Insulin-like growth factor signalling is known to be pro-proliferative on fibroblast cells of different origin 29,30. This was found to be by increasing the expression of the IGF-II ligand in these cells. In congruence with this, OSF tissues also have elevated levels of IGF-I and II ligands. These data suggest involvement of IGF-signalling in areca nut pro-proliferative effect on fibroblasts. Our results are also in agreement with the previous observations suggesting proliferative effects of arecoline and arecaidine on fibroblasts 31. Therefore, proliferation of fibroblasts involving IGF signalling mediated by areca nut components could be one of the important events in the OSF pathogenesis.

### Areca nut induced cytotoxicity of keratinocytes

Epithelial atrophy is one of the hallmarks of OSF. Thus far there have been no conclusive studies that provided a mechanistic explanation for this event. Several possible reasons for epithelial atrophy could be envisaged that include cell death, loss of proliferative capacity, loss of the stem/progenitor population *etc*. We addressed these possibilities by treating the keratinocytes with areca nut components. As presented in the results, we demonstrated apoptosis of keratinocytes upon treatment with areca nut extract. By a variety of approaches, we demonstrated that this effect is ROS dependent. We also demonstrated arecoline as a principal mediator of this effect. Our results are in line with the literature where arecoline was found to be cytotoxic on different cell types, possibly mediated by ROS 4,20,32. Intriguingly, the IC_50_ of areca nut extract was much lower than the IC_50_ of arecoline, suggesting the role of other components in mediating areca nut cytotoxicity along with arecoline. Our experiments revealed copper being a significant component of areca nut potentiated the arecoline mediated cytotoxicity. This was substantiated by physical studies, such as cyclic voltammetry, UV spectrophotometry and plasmid cleavage assay, which showed increased oxidation of copper (II) in presence of arecoline leading to cleavage of DNA in a superoxide dependent manner. This induced DNA cleavage by arecoline and copper (II) is also supported by their complex formation as shown by the mass spectrometry studies (ESI-MS). Accordingly, like areca nut, cytotoxicity induced by the arecoline along with copper also follows apoptotic pattern of cell death which is dependent on ROS generation. It is important to mention that areca nut alkaloids also form *N*-nitroso-compounds after their metabolism (like NMPN, 3-(*N*-nitrosomethylamino) propionitrile), which are highly cytotoxic 20 and may also contribute to its cytotoxicity *in vivo*. In contrast, areca nut extracts did not show induction of ROS in fibroblasts and this could be associated with higher expression of catalase and heme oxigenase that are ROS neutralizing enzymes. Based on these results we propose that inherent differences between epithelial and fibroblast cells could make them sensitive or tolerant to areca nut induced cytotoxicity.

### Role of cytotoxicity in the epithelial atrophy of OSF

As discussed before areca nut is cytotoxic to epithelial cells and causes apoptosis. However, cell death/apoptosis has not been demonstrated in OSF tissues. We hypothesized that as the oral tissues of areca nut chewers are exposed for prolonged duration with areca nut; their epithelium might be under oxidative stress and could be dying of apoptosis. Our experiments revealed higher TUNEL positivity in OSF tissues compared to normal tissues. Furthermore, Ki-67 LI was much higher in those OSF patients where TUNEL positivity was also high suggesting that these tissues have higher proliferation index. But Ki-67 and TUNEL positive cells were observed on opposite layers of epithelium namely, basal epithelial cells and keratin or *supra* basal epithelial cells, respectively. This induced proliferation of epithelial cells could be due be a compensatory mechanism to cope up with the significant apoptosis in the outer epithelium. Alternately, the increased Ki-67 index in the inner epithelial layers could be due to the action of pro-proliferative growth factors secreted by the stromal cells. As discussed in the previous section, areca nut activates IGF signalling by the induction of IGF-II in fibroblasts. Although areca nut does not induce IGF ligands 6 in epithelial cells, these cells may respond to the stromal IGFs 33,34. One could ask a question as to why stromal IGFs does not protect the outer epithelial layers from apoptosis. For this, we speculate that areca nut induced cytotoxicity in epithelium is considerably higher, and growth factors secreted by the stromal (hGF) cells are not adequate to provide a survival benefit to the far off epithelial layers and hence leading to atrophy.

The proliferative index could also be a sign of precancerous change. This needs further exploration. Since these patients are regularly exposed to areca nut, we believe that this increased proliferation may not be able to compensate for the apoptosis induced by areca nut, hence leading to atrophied epithelium. One other possibility for the epithelial atrophy could be the status of stem cells in the tissue. It is well-known that any tissue regeneration or repair depends on the respective stem cell pool in that tissue. Depletion or failure of proliferation of stem cells in the epithelium could be a possibility for epithelial atrophy in OSF. Our preliminary observation based on Oct4 immunostaining of OSF tissues suggested fewer OCT4 positive nuclei in the epithelium of majority of OSF patients as compared to OCT4 positive cells in the epithelium of all normal tissues (data not shown). Interestingly there are no reports we could find that studied stem cell status in OSF. Further experiments using multiple stem cell markers on a larger cohort of samples are necessary for any conclusive inference. Therefore, both cell death and possible loss of stem cells could be the compounding factors in the epithelial atrophy of OSF patients.

In conclusion, we show pro-proliferation and cytotoxicity as two differential effects of areca nut on fibroblast and epithelial cells, respectively. We also provide a mechanism of areca nut cytotoxic actions involving complex formation between arecoline and copper that may explain the epithelial atrophy in the development of OSF. These actions of areca nut along with its role in inducing epithelial factors (pro-fibrotic factors such as TGF-β) 6 may have an additive/synergistic effect in OSF initiation/potentiation.

## Supporting Information

**Figure S1** Pro-proliferative effect of areca nut extracts by BrdU correlates with IGF pathway activation.

**Table S1** List of primer sequences used for RT-PCR.

**Table S2** Labelling index of Ki-67 and TUNEL in OSF and normal epithelial cells.
