# Supplementary figures and images for: Epithelial atrophy in oral submucous fibrosis is mediated by copper (II) and arecoline of areca nut
Source: J Cell Mol Med. 2015 Aug 6;19(10):2397–412. doi: 10.1111/jcmm.12622 (PMC4594681; doi:10.1111/jcmm.12622)

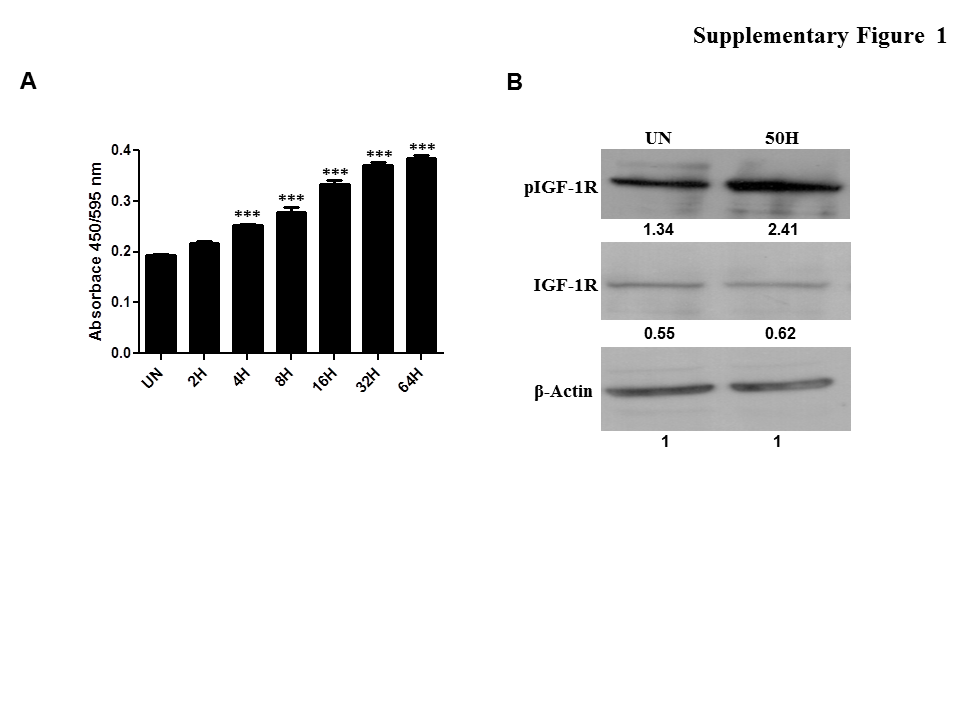

Supplement: Supplementary file 1 [file jcmm0019-2397-sd1.tif]
