# Supplementary material for: Epithelial atrophy in oral submucous fibrosis is mediated by copper (II) and arecoline of areca nut
Source: J Cell Mol Med. 2015 Aug 6;19(10):2397–412. doi: 10.1111/jcmm.12622 (PMC4594681; doi:10.1111/jcmm.12622)
Supplement: Supplementary file 2 [file jcmm0019-2397-sd2.doc]

**Table S1: List of primer sequences used for RT- PCR**

| **Sl.No** | **Genes** | **F 5’-3’ sequence** | **R 5’-3’ sequence** | **Description** |
| --- | --- | --- | --- | --- |
| 1 | HOX-1 | ATGACACCAAGGACCAGAGC | GTGTAAGGACCCATCGGAGA | 158 bp, 58°C |
| 2 | IGF-I | TCAGAAGCAATGGGAAAAATCAGC | TCCTTAGATCACAGGTCCGGAAGC | 319 bp, 60°C |
| 3 | IGF-II | GCTTCCAGACACCAATGGGAATCC | TCATATTGGAAGAACTTGCCCACG | 364 bp, 60°C |
| 4 | RPL35A | GAACCAAAGGGAGCACACAG | CAATGGCCTTAGCAGGAAGA | 236 bp, 58°C |
