# Supplementary material for: Epithelial atrophy in oral submucous fibrosis is mediated by copper (II) and arecoline of areca nut
Source: J Cell Mol Med. 2015 Aug 6;19(10):2397–412. doi: 10.1111/jcmm.12622 (PMC4594681; doi:10.1111/jcmm.12622)
Supplement: Supplementary file 3 [file jcmm0019-2397-sd3.doc]

**Table S2: Labeling index of Ki-67 and TUNEL in OSF and normal epithelial cells.**

| **Normals** | **LI (Ki67 )** | **Epithelial layer** | **LI (TUNEL)** | **Epithelial layer** |
| --- | --- | --- | --- | --- |
| N1 | 8 | Basal cells | 0 | NS |
| N2 | 10 | Basal cells | 1 | Keratin, Supra basal |
| N3 | 17 | Basal cells | 1 | Keratin, Supra basal |
| N4 | 17 | Basal cells | 1 | Keratin, Supra basal |
| N5 | 3 | Basal cells | 1 | Keratin, Supra basal |
| N6 | 5 | Basal cells | 1 | Keratin, Supra basal |
| N7 | 14 | Basal cells | 0 | NS |
| N8 | 8 | Basal cells | 1 | Keratin, Supra basal |
| N9 | 16 | Basal cells | 2 | Keratin, Supra basal |
| N10 | 13 | Basal cells | 1 | Keratin, Supra basal |
| OSF1 | 0 | NS | 0 | NS |
| OSF2 | 58 | Basal cells | 54 | Keratin, Supra basal |
| OSF3 | 0 | NS | 0 | NS |
| OSF4 | 1 | Basal cells | 18 | Keratin, Supra basal |
| OSF5 | 38 | Basal cells | 25 | Keratin, Supra basal |
| OSF6 | 21 | Basal cells | 37 | Keratin, Supra basal |
| OSF7 | 14 | Basal cells | 1 | Keratin, Supra basal |
| OSF8 | 8 | Basal cells | 12 | Keratin, Supra basal |
| OSF9 | 5 | Basal cells | 4 | Keratin, Supra basal |
| OSF10 | 33 | Basal cells | 60 | Keratin, Supra basal |
| OSF11 | 0 | NS | 0 | NS |
| OSF12 | 33 | Basal cells | 49 | Keratin, Supra basal |
| OSF13 | 23 | Basal cells | ND | ND |
| OSF14 | 26 | Basal cells | 0 | NS |
| OSF15 | 0 | NS | 0 | NS |
| OSF16 | 10 | Basal cells | 7 | Keratin, Supra basal |
| OSF17 | 1 | Basal cells | 7 | Keratin, Supra basal |

(NS- Not stained, ND- Not determined)
